# Proteinquakes in the Evolution of Influenza Virus Hemagglutinin (A/H1N1) under Opposing Migration and Vaccination Pressures

**DOI:** 10.1155/2015/243162

**Published:** 2015-01-13

**Authors:** J. C. Phillips

**Affiliations:** Department of Physics and Astronomy, Rutgers University, Piscataway, NJ 08854, USA

## Abstract

Influenza virus contains two highly variable envelope glycoproteins, hemagglutinin (HA) and neuraminidase (NA). Here we show that, while HA evolution is much more complex than NA evolution, it still shows abrupt punctuation changes linked to punctuation changes of NA. HA exhibits proteinquakes, which resemble earthquakes and are related to hydropathic shifting of sialic acid binding regions. HA proteinquakes based on shifting sialic acid interactions are required for optimal balance between the receptor-binding and receptor-destroying activities of HA and NA for efficient virus replication. Our comprehensive results present a historical (1945–2011) panorama of HA evolution over thousands of strains and are consistent with many studies of HA and NA interactions based on a few mutations of a few strains.

## 1. Introduction

A previous paper [1] showed that punctuated evolution and strain convergence occur in NA1 (H1N1) and can be identified by studying amino acid mutational changes of strains in Uniprot and NCBI databases. The punctuations arise because of migration (interspecies, avian, swine,…, or geographical jumps), which can increase antigenicity and large-scale vaccination programs, which not only reverse migration effects, but also go further and have led to large overall antigenic reductions. It turned out that it was easy to monitor all these effects on NA simply by studying the global roughness of water-NA chain interfaces, using hydropathic methods previously applied to other membrane proteins, such as rhodopsin [2, 3]. The success of these methods was greatly improved by the use of the MZ hydropathicity scale based on self-organized criticality (SOC) [4]. SOC explains power-law scaling, and it is arguably the most sophisticated concept in equilibrium and near-equilibrium thermodynamics. This result shows a gratifying effective internal consistency, as SOC arises because of evolution, and its success in recognizing and quantifying punctuated evolution and strain convergence shows that long-range hydropathic interactions can dominate the more familiar short-range ionic and covalent interactions, which are the only ones treated by other methods, in optimizing evolving interactions of sufficiently large proteins.

How large must a protein be to be “sufficiently large” for long-range forces to dominate its functionality? Methods based on short-range interactions (van-der-Waals, hydrogen bonding, and continuum solvation “effective water”) within Euclidean structures have emphasized chemical-protein epitope interfaces that are usually limited to 15–20 amino acids, although recently these have been expanded in yeast-based deep sequencing studies to 50 amino acids [5]. Because the hydropathic interactions between water films and protein substrates are weak, it has traditionally been assumed that evolutionary energy changes in short-range chemical interaction energies always dominate long-range hydropathic energy changes.

Short-range packing interactions die out for amino acid sequences longer than 9, leaving mainly water-protein roughening interactions at longer range. The packing interactions are so strong that the energies associated with them change little with tertiary conformations. The long-range dominance of hydropathic interactions is the reason that the evolution of solvent accessible surface areas follows power laws for each centered amino acid in segments of length 2*N* + 1  (4 < *N* < 17) in the bioinformatic survey of 5526 PDB segments, as expected from SOC and Darwinian evolution [4, 8].

**Figure 1:**
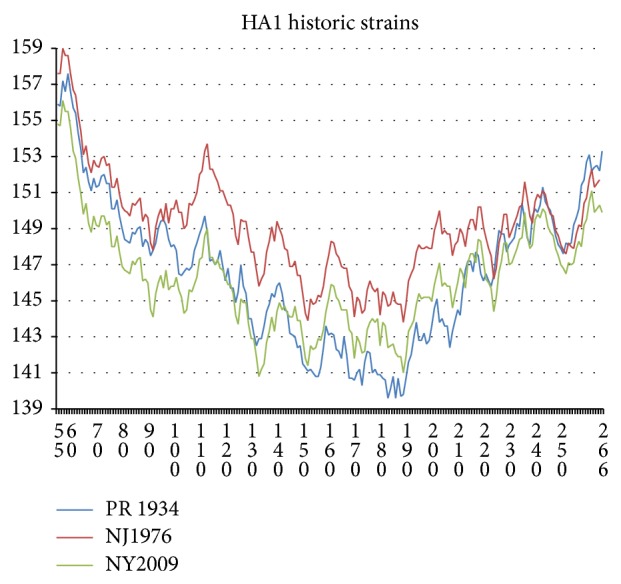
HA (1–565) is structurally separated into head HA1 (18–342) and stalk HA2 (344–565) parts, the latter being attached to the membrane. The head hydroprofiles of the two historic HA strains, Fort Dix 1976 (Genbank ACU80014) and Puerto Rico 1934 (P03452), are compared with a modern (vaccination moderated) strain, New York ACQ84467. The origin of the severity of the 1976 Fort Dix strain is explained by its strongly hydrophobic head amino acid content, especially near site 110. The advent of swine flu (2001–2007) and the response (2009–2011) to its vaccination program flattened (smoothed) current strains (like New York 2009) and reduced their virulence.

As noted in [1], most of the evolutionary changes in HA occur in the head chain HA1 (18–342) denoted by HA1. As discussed in more detail below, these changes are best displayed with hydropathic chain profiles using the MZ scale [4] averaged over a long sliding window length *W* = 111. On an historic scale, going back to the 1918 pandemic, these HA1 changes are large and dramatic, as shown in Figure 1.

**Figure 2:**
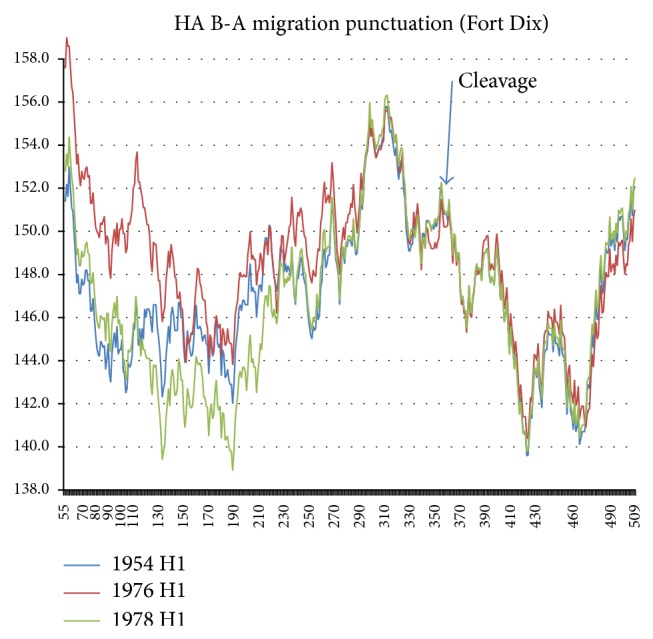
The full 〈*ψ*(*j*)111〉 chain profiles of three HA sequences, Netherlands 1954, Fort Dix outbreak (1976), and postvaccination California 1978. The cleavage site separates HA1 from HA2. Mutations occur primarily in the HA1 region below 300. Note that the “highly conserved H1 subtype-specific epitope” [6] 58–72 became strongly hydrophobic in the Fort Dix outbreak. The epitope covering 108–122 [6] includes the spectacular Fort Dix peak near site 117. Studies with the epitope covering 108–122 of mice infected with the three strains shown here would be well worth further effort.

**Figure 3:**
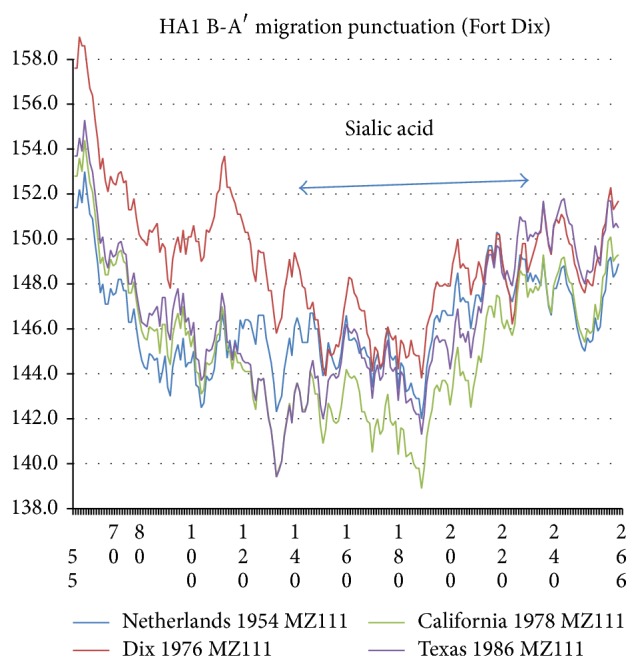
The 〈*ψ*(*j*)111〉 chain profiles of four HA1 sequences, Netherlands 1954 (ADT78876), Fort Dix outbreak 1976 (ACU80014), postvaccination California 1978 (ABY81349, identical to Memphis 1983), and Texas 1986 (ABO44123), cut off at 266 (A1 chain only) to show more clearly mutational trends below 220. The Fort Dix outbreak increased hydrophobicity, especially below 150. After the 1976-77 vaccination program, in 1978–86 hydrophobicity dropped below the 1954 level in the region 120–230, spanning the two sialic acid binding sites determined crystallographically [7].

The 565 amino acids of compacted HA exhibit a rich Euclidean structure composed of two chains, HA1 and HA2 [9]. The two ends of the globularly combined chains are stabilized by hydrophobic peaks, and there is a third peak near 310 stabilizing the center of HA. See Figure 2, which exhibits changes in the hydropathic chain profile using the MZ scale [4] averaged over a long sliding window length *W* = 111; this choice of *W* will be discussed below. The various evolutionary punctuations of companion NA sequences [1] will be examined here for HA (Figure 2 shows one of them, the 1976 Fort Dix outbreak, accompanied by a hasty vaccination program.) The central third 310 hydrophobic peak occurs on the HA1 side of the 343 cleavage site. It stabilizes HA1 after the fusion segment 345–367 of HA2 merged with the target membrane. As in Figure 3 almost all the evolutionary changes occur in HA1 1–300, and this is the region discussed here in detail (see Figure 3). It is dominated by the globular head domain, which includes the sialic acid binding site 130–230 and a number of glycosylation sites, which have increased since 1918 [9].

Uniprot lists nine glycosylation sites in A3DRP0, Memphis/10/1996 HA1 (27, 28, 40, 71, 104, 142, 177, 286, and 304) and only one on HA2 (498). Glycan microarray analysis has revealed glycosylation differences between either remote H1N1 strains (1918, modern) at a few sites [10] or differences between H1N1 and other subfamilies such as H5N1 [11]. The multiple glycosylation sites apparently contribute mainly to the HA agglutination function, as will be seen below.

Before we leave the 1976 New Jersey outbreak, we can use it to make a simple connection between HA and NA mutations. As noted in the abstract of [1], the mutational changes to NA are much smaller than those to HA. This means that it is much easier to measure differences in properties due to antigenic drift for HA than for NA, and experimentalists will focus their efforts on HA. However, an accurate theory will yield its clearest results for the simpler case of NA, which is why NA was treated first in [1].

There are about 30 HA 1934 Puerto Rico sequences in Genbank, with an overall 99% identity. If we use BLAST to track subsequent evolution of these sequences (for instance, AFM71846, or the Mt. Sinai sequence AAM75158), we find a few intermediate sequences from Melbourne (1935, 94% and 1946, 93%) and then 1947 New Jersey (90%) and 1976 New Jersey (85%). Thus (as expected) there has been much more HA drift than NA drift. However, much of the HA drift is due to frequent aa mutations, so that HA positives (as defined by BLAST) are still 91% between 1934 Puerto Rico and 1976 New Jersey.

## 2. Methods

The methods used here are almost the same as these used in [1] for NA but with a few important changes. The evolution of NA was monitored by calculating the roughnesses of *R*_KD_(*W*_max⁡_) and *R*_MZ_(*W*_max⁡_) with *W*_max⁡_ = 17 of the water packaging film with two scales, KD and MZ. These roughnesses (especially with the MZ scale) showed well-defined plateaus connected by punctuated decreases (vaccination programs) or increases (migration). (The MZ improvement is clear even though the overall correlation of *R*(17) for NA for the two scales is around 90%.) The superior NA resolution of the MZ scale has led us to report HA results here only for the MZ scale. The sliding window width used for calculating the NA roughnesses was set at *W*_max⁡_ = 17, although 21 (closer to the transmembrane thickness) would have been equally good. This width was also used in displaying chain hydroprofiles 〈*ψ*(*j*)*W*〉. Note that *W* functions as a modular length, which appears naturally in the model by optimizing its resolution.

**Figure 4:**
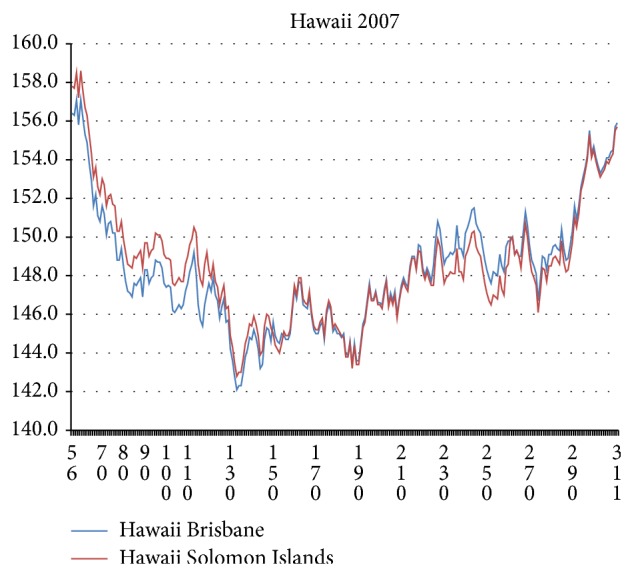
The 〈*ψ*(*j*)111〉 HA1 chain profiles (55 < *j* < 311) of Genbank Brisbane ACB11812 (B) and Solomon Islands ACA33672 (A) [Hawaii 2007]. The key B-A mutations are K64I, MT (205, 206) KA, and N238D. The left-right difference crossover near 180 can be described as a hydropathic twist.

The historical (1945-) directed evolution of roughnesses towards smaller values found for NA with *W* = 17 does not occur for HA. Instead, at each punctuation of NA, the HA chain hydroprofile 〈*ψ*(*j*)*W*〉 exhibits hydrophobic stabilization over selected blocks whose edges tend to coincide with one or both of the edges of the sialic acid binding site 130–230. Because this site is so wide, we have chosen to display hydroprofiles 〈*ψ*(*j*)*W*〉 with *W* = 111. To test this choice, we selected several HA sequences from Hawaii 2007. As was shown in [1], Hawaii 2007 consists mainly of two hydropathically recognizable subsets, one labeled “Brisbane,” and the other, “Solomon Islands.” The differences in their NA *R*_MZ_(17) are large (30% of the historical shift from strain A to strain D^*^) [1], while their HA *R*_MZ_(1) differences are only 1.5% of *R*_MZ_(1), which is only 2∑, where ∑ is the sum of the *σ*'s of each subset [1]. From HA Hawaii 2007 we selected two sequences (ACB11812, Brisbane and ACA33672, Solomon Islands) with the largest BLAST nonpositive differences and the largest *R*_MZ_(1) differences. Their 〈*ψ*(*j*)111〉 chain profiles exhibit a striking sign difference reversal near 180, presumed to be associated with a change in sialic acid binding (Figure 4). A smaller window value of *W* = 75 shifts the crossover to below 180 and introduces secondary sign reversals, apparently associated with inadequate sliding window resolution (essentially Fresnel fringes associated with hydropathic waves at the sharp sialic acid edges). This shows that *W* = 111 is an excellent choice for HA1 sliding window width, as expected from its similarity to the length of the sialic acid binding site. An interesting point is that there is excellent correlation (87%) of *R*(111) for HA1 for the MZ and KD scales for the period 1918–2001, but this correlation breaks down with the advent of swine flu.

The large value of *W*, as well as the dominance of overall interactions with sialic acid on the length scale *W* ~ 111, explains why the details of glycosylation interactions on the length scale of glycosylation spacing (three times smaller) are important only for large-scale strain differences. A survey of 50 immunodominant epitopes (typically 15 aa long) spanning HA Calif 04/2009 revealed a distinct subset that had nearly equal autoantibody interactions almost twice as strong as the remaining epitopes [12]. There are four epitopes in this remarkable subset. As expected, all of these four occurred among the 34 epitopes in the head region below 350. The single criterion that these four (about 10% of all head epitopes studied) satisfied is that they are all either in hydrophobic or hydrophilic extrema (see also Figure 10). The likelihood of such a subset occurring accidentally is <10^−7^. Note that the strong interactions were equally strong for all four cases. This is what one expects, as the two kinds of extrema are weighted equally in calculating the variance, which does not depend on the sign of the deviation from average. However, the equal weighting is surprising in Euclidean terms, as the hydrophobic/hydrophilic epitopes are on the inside/outside of the globular cluster, and inside/outside are not obviously equivalent. Finally, one of the sites identified as significant by glycan microarray analysis [10] is 190, which is seen in the chain hydroprofiles (Figure 3) as one of several hydrophilic extrema.

## 3. Results

**Figure 5:**
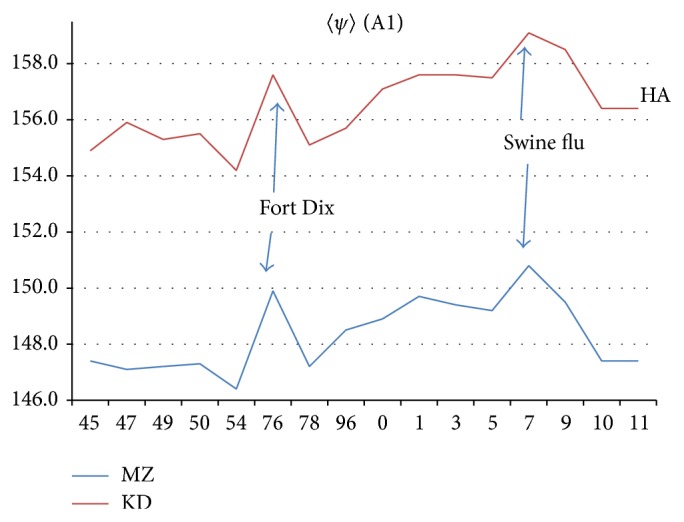
Sketch of chain A1 〈*ψ*(aa, 1)〉 evolution, with Fort Dix outbreak and swine flu peak (before vaccination program) indicated. The HA 〈*ψ*(aa, 1)〉 trends seen here parallel those for NA tabulated in [5].

Perhaps the simplest difference between HA and NA profiles is the fact that NA profiles have overall *ψ*_MZ_ averages = 〈*ψ*_MZ_〉 nearer 155 (hydroneutral, which is normal) than HA. This is shown in Table 1, averaged over selected strains. For NA we estimated geographical scatter *σ* from Hawaii 2011. Here for HA we find systematic parallel motion of 〈*ψ*_MZ_〉 and 〈*ψ*_KD_〉 with peaks at Fort Dix (New Jersey 1976) and swine flu (2009), as shown in Figure 5. These correlate with the punctuations found in NA and have a natural interpretation as the HA analogs of the NA flu pandemics, which were explained in the NA paper. Thus we can estimate the HA intrinsic scatter by evaluating 〈*ψ*_MZ_〉 and 〈*ψ*_KD_〉 in a year where there was little activity (we used 1996) and using strains widely dispersed geographically.

In Figure 5 we can compare Table I's *σ* with the Fort Dix outbreak hydrophobic peak and successive outbreaks of the swine flu pandemic (which appeared in Brazil 2001 and then grew in New York 2003, and Berlin 2005, and finally reaching a peak in Texas 2007), we find that the 〈*ψ*〉 peaks are *ασ* above background, with *α* ~ 4 for the MZ scale and ~3.5 for the KD scale. This is certainly an impressive resolution, but can we not do better? Can we not connect these average changes to the chemistry of HA?

We can. We return to Figure 2, which shows that both the A1 and A2 chains of HA are predominantly hydrophilic, with deep minima near 140. Even the relatively hydrophobic stabilizing peak near site 310 peaks near only 156 (hydroneutral). At present the reasons for this exceptionally hydrophilic HA character are not clear, but there are several possibilities. Strong interactions with water are the result of larger surface/volume ratios, and larger surfaces could facilitate cylindrical oligomer formation. This could explain why engineered HA sequences and not NA sequences have been used in vaccines, where they could block oligomer formation [6, 13, 14].

The expanded 〈*ψ*(*j*)111〉 HA1 chain profiles for the 1976 migration-vaccination punctuation are shown in Figure 3. The large HA shift of the New Jersey (Fort Dix outbreak) sequence is expected; the Fort Dix outbreak included a large increase in the NA *R*_MZ_(17) roughness and an even larger increase for *R*_KD_(17) [1]. More interesting is the NA shift from 1954 to 1978–1986: superstrain B (1954) reverts to superstrain A (1978–1986) so far as NA roughness is concerned (Table I of [1]). Here it is seen that HA progresses and becomes more flexible than NA, as the sialic acid region 120–230 becomes more hydrophilic (softer and more open) in 1978–86 than in 1954. Between 1954 and 1978 there are 10% nonpositive mutations in HA1, so the effects of the Fort Dix outbreak on HA were large. There are fascinating details in the figure. For example, in 1978 we see two sharp hydrophilic minima at 130 (sialic acid site edge) and 190 (sialic acid site center), with the latter weakening by 1986. What happened from 1978 to 1986 was the KDQKTIYQK (203–211) GDQRAIYHT mutation, which concentrates four nonpositive mutations (KG, TA, QH, and KT), all four increasing hydrophobicity cooperatively and closing (tightening or compacting) the structure.

The first punctuation (A-B) of NA *R*_MZ_(17) occurred in connection with the vaccination program of the American army began in 1944, and its benefits were largest in the Netherlands 1954 sequence [1]. For both NA and HA the punctuation itself was most apparent in the Rome 1949 Ft. Warren 1950 sequences, as shown in Figure 5. The largest effect was the hydrophobic plateau that occurred in the sialic acid receptor central block between 110 and 230 (sialic acid binding) in Ft. Warren 1950, but it was preceded by a smaller hydrophobic increase in the N terminal block below 130 in Rome 1949, which in turn was preceded by a still smaller hydrophilic increase in the P terminal block above 230. After the initial 1950 punctuation, the entire chain profile reverts in 1954 to nearly its 1945 state, except that there is weak hydrophilic softening of the terminal sialic acid matrix blocks below 110 and above 230.

**Figure 6:**
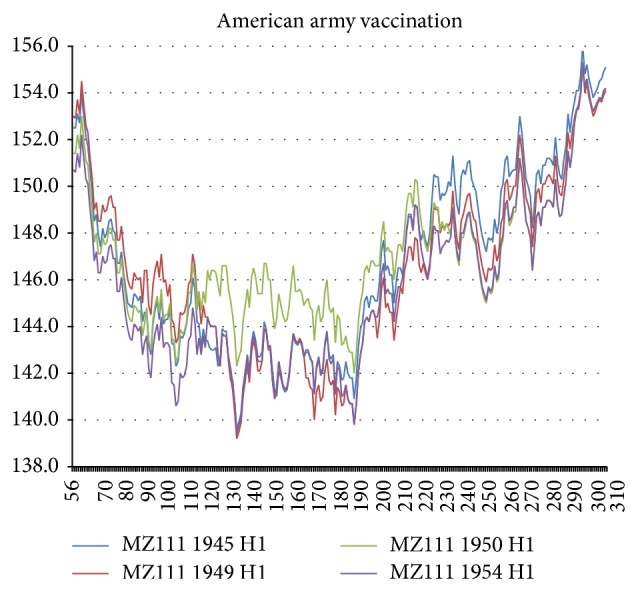
The army vaccination punctuation *W* = 111 (MZ) chain profiles resemble an earthquake with respect to the sialic acid binding site 130–230 relative to its HA1 matrix (see text). Note that the main feature of the sialic acid binding site is not only the hydrophilic extrema at its end points, but also the flattening of the HA butterfly profile across the entire 130–230 binding range, especially in 1950. This reflects the strongly one-dimensional nature of network water film packages, which is not obvious in Euclidean simulations of protein dynamics using effective water models.

What does this precursive pattern mean? It is mysteriously similar to an earthquake with a precursor and an aftershock, but that is not so mysterious after all (Figure 6). If proteins are indeed near self-organized critical states, then they might well exhibit tertiary hydropathic shocks resembling template earthquakes, as one of the first (and still perhaps the most popular, 640 papers) applications of SOC has been to earthquakes [15–17], where collisions of tectonic plates are described by spring-block models [18]. These quakes occur in the water film packaging HA1 and resemble earthquakes in the earth's crust. Growing actin networks also exhibit sporadic effects predicted by SOC [19]. These structural changes can be described as proteinquakes.

**Figure 7:**
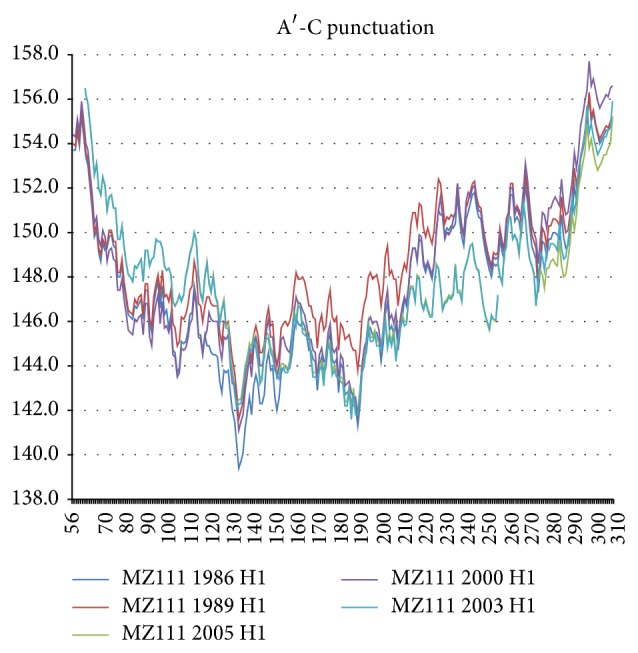
During the period 1983–2005 NA exhibited a plateau that terminated with punctuation B-C (advent of swine flu), which occurred in Brazil (AAY42117) in 2001, in New York in 2003, in Berlin in 2005, and in Texas in 2007. The advent of swine flu also caused the HA1 block below 130 to increase hydrophobically almost identically in New York in 2003 and in Berlin in 2005, as shown here. The Genbank sequences used here are ABO44123 (Texas 1986), ACL 12261 (Siena 1989), ACI32714 (Berlin 2005), AAX56530 (New York 2000), and ABB82205 (New York 2003).

**Figure 8:**
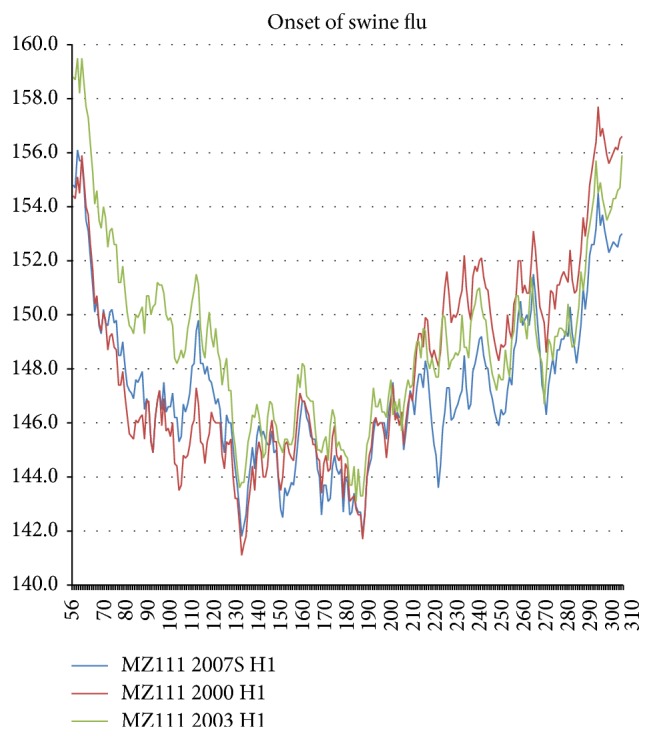
Comparison of human strains from New York 2000 and 2003 with swine flu from Kansas 2007. The similarities are obvious, but what actually happened is described in Figures 9 and 10.

The 1989–2003 NA smoothing gain ended with the advent of the “swine flu” strains, which appeared in successive outbreaks, first in Hong Kong 1999 and next in New York in 2003 and in Berlin in 2005. The characteristic HA feature of these strains was a large hydrophobic increase in the N terminal sub-130 block, which was almost identical for New York and Berlin (Figure 7). These hydrophobic block increases correspond to block compressive elastic stiffening. As shown in Figure 8, comparison with an actual 2007 swine flu sequence shows that the latter sequences were also evolving rapidly. How did flu evolution in swine compare with human flu evolution?

**Figure 9:**
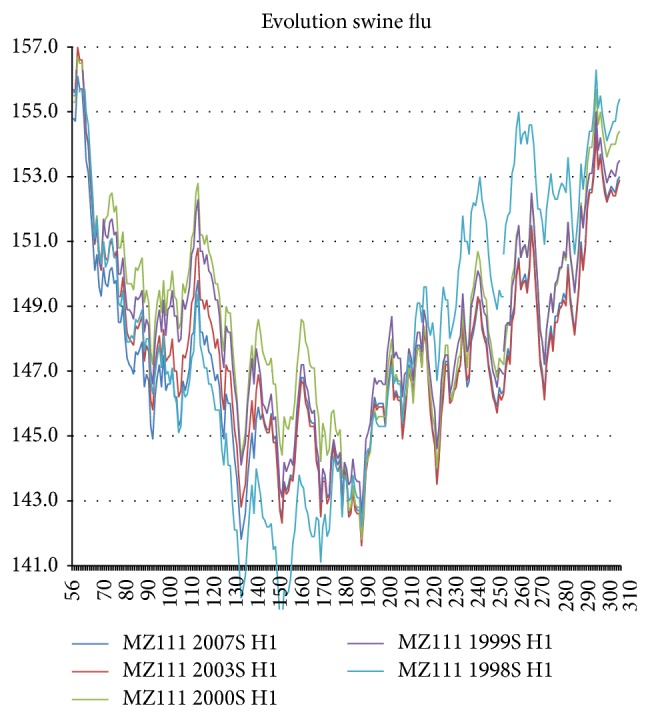
The HA1 swine flu sequences profiled here are England 1998, Hong Kong 1999, North Carolina 2000 and 2003, and Kansas 2007.

As shown in Figure 9, a new superstrain of swine flu first appeared in Hong Kong in 1999. The England 1998 strain was very different, being much more hydrophobic in the post-230 block and much more hydrophilic in the silaic acid block 130–230 (presumably less antigenic). Hong Kong 1999 swine flu quickly spread to North Carolina 2000, but by 2003 its increased 130–230 antigenicity had been halved, and the increase in the sub-130 block had nearly disappeared. Thus the new flu strain appeared in swine several years before its first human appearance in New York in 2003 and had been controlled by vaccination in swine before the new strain had become a human problem, which probably limited its human impact.

**Figure 10:**
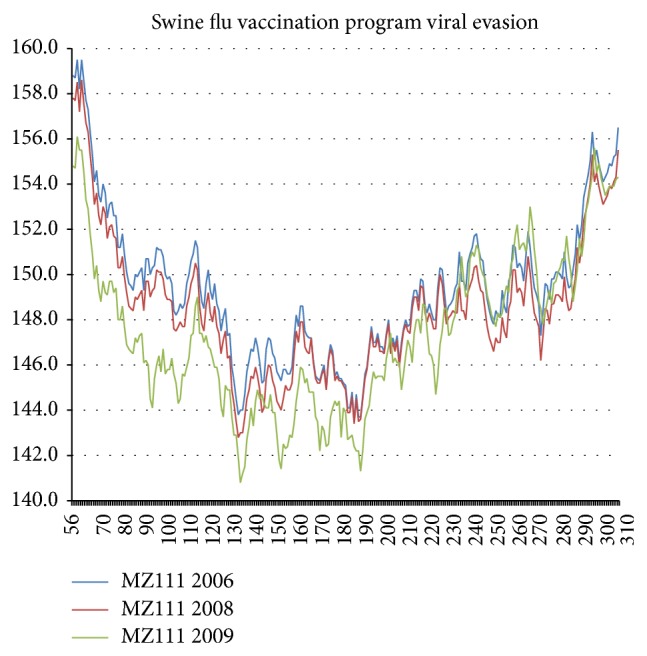
North Carolina response to swine flu vaccination program, which began in 2007. The small response in 2008 is greatly enhanced by 2009, after which changes were minimal. The key mutations from 2008 (ACD45795) to 2009 (ADM21399) often involve not individual sites but short strings, for instance, TATY13-16ATAN, LLISKE86-91SLSTAS, and TVT144-147DSNK, indicative of both long-range hydrophilic softening and short-range expansion, including the hydrophilic insertion 144D. The strongly antibody producing epitopes for California 2009 [6] were all at HA1 hydropathic maxima (38–73), (318–332) or minima (158–182). Thus the hydroprofile shown here is more informative than the Euclidean structural illustration shown as an inset to Figure 2 of [6].

With a larger data base, we can study the response to the 2007 human swine flu vaccination in one locality, which was done for North Carolina and for Norway. The results for North Carolina (see Figure 10) show a large shift towards hydrophilicity between 2008 and 2009. The results for Norway are similar but with an interesting difference. In 2006 there were 4 nonpositive BLAST mutational differences between North Carolina and Norway, but by 2009 the two sequences had converged to become identical. This convergence probably occurred as a result of viral evasion of a common vaccination program, which was so effective as to erase substantial viral climatic differences. A simple search of the “identical proteins” feature of the HA NCBI data base, similar to the previous one for NA [1], is less rewarding for HA because of a wider range of reported HA lengths. However, one length (566 aa) is sufficiently common in the 2009 and 2010 HA data to be indicative of effects similar to those found for NA: a single very common strain in 2009, which becomes less common in 2010 as vaccination pressures receded. According to [1], the effects of the swine flu vaccination program shown there on human NA are remarkable, as the human strains made a very large “Lévy” jump from superstrain C to superstrain D (not seen in earlier punctuation small jumps) to avoid the vaccine and dodge swine flu, while reducing their severity. Apparently the large hydrophilic softening seen in Figure 9 is a Lévy effect for HA. These two large Lévy effects in sequence space, which describe explicitly smoothing of NA and block softening of HA, are quantitative measures of the optimal balance between receptor-binding and receptor-destroying activities of NA and HA that is required for efficient virus replication [20].

These large jumps in HA block hydropathicity are mediated by strings of single mutations, which were not seen in NA. Also NA contracted slightly and stiffened in response to the swine flu vaccination program, while HA expands and softens, another example of NA-HA “balance” [20]. One is not surprised to find that HA has a block structure, because it binds to the cell that is being infected through sialidase, which is a large molecule. Also it seems natural that the large number of HA mutations induced by the human swine flu vaccination program should occur in short strings (see figure captions), rather than as largely isolated single mutations (as in NA), as this is a more effective way to alter block hydropathic structure, with fewer changes in short-range packing.

**Figure 11:**
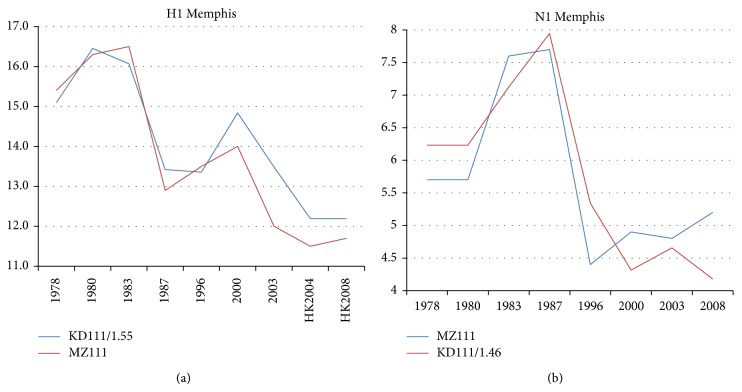
Broad trends in *W* = 111 roughnesses for (a) HA1 and (b) NA. Data primarily from Memphis.

Could HA *W* = 111 block hydropathic roughnesses have a secondary effect on NA at *W* = 111? This question is addressed in Figure 11, with data restricted to Memphis. We see in (a) a broad downward trend in HA1 with two peaks in 1983 and 2000, but these either follow the 1976 *W* = 17 NA peak or precede the 2007 swine flu peak. In other words, HA evolution may be coupled to NA evolution but not in a simple way. This point is brought out in (b), which shows only one peak, placed between the two HA peaks. There appears to be some measure of complementarity of HA and NA evolution on the sialidase *W* = 111 scale. Overall we recognize that H1N1 HA and NA evolution is dominated by at most two length scales associated with N-glycans and sialic acid. Because our analysis is hierarchical (it distinguishes between increasing and decreasing roughness) and includes detailed quantitative aspects of sequence evolution on large length scales not accessible to similarity analysis, it could be useful in refining binding data [5, 21]. Our method is effective over long length scales not accessed by methods used to study short synthetic peptides [22].

## 4. Discussion

Both here and in our previous paper on NA [1], we have shown that the vaccine developed to prevent swine flu has been extremely effective. Most recent discussions of H1N1 have focused on studying the cellular origins of this positive response, a kind of vaccine windfall. The response has been attributed to broadly cross-reactive antibodies generated by B cells [23], but of course these in turn have been induced by appropriate vaccines [7, 24]. Here and in [1] we have shown that this recent windfall, suggestive of a “universal vaccine,” is one part of a vaccination-driven panorama whose origins for H1N1 can be traced back to the original vaccination programs which began in 1944. The distinctive feature of our analysis is that it quantifies all these beneficial effects using sequence-specific tools that can be applied to discuss universally all proteins and their interactions. Specifically the similar effectivenesses of the 2009 and 1976 vaccines are expected from Figure 6 of [1] and from Figure 5 here.

## 5. Conclusions

The analysis of NA using hydropathic roughness with *W* ~ 20 as a configuration coordinate revealed opposing punctuations due to migration (increasing roughness) and vaccination programs (increasing smoothness) [1]. Here we have found that balanced interactions between HA and NA also cause parallel punctuations in HA at the same times. However, the HA punctuations are more complex and require studying hydropathic chain profiles with large sliding window lengths of order the spacing between the sialic acid binding sites, *W* ~ 100. The HA1 profiles have a characteristic broad and deep (V-shaped or butterfly) hydrophilic minimum in the sialic acid binding region 130–230 and are strongly hydrophilic. The overall evolution of HA1 shows primarily shifts in hydropathicity of three chain blocks, 50–130, 130–230, and 230–300. These punctuated block shifts closely resemble earthquakes [15–17] in one-dimensional sequence space, consistent with analyzing them using the MZ hydropathicity scale based on self-organized criticality. Unlike NA roughness shifts, which are often associated with a few single amino acid mutations, HA block shifts are often associated with sequences of up to 9 amino acids, which reflect the epitopic chemistry [12] of short-range HA-sialidase interactions. The hydropathic chain profiles can be used as biomarkers to represent HA-sialidase interactions.

The central limitation of prior studies of viral kinetics has been their low resolution, limited to the large N-glycan spacing length scale [10, 11] or the even larger sialic acid length scale [25]. There one finds evidence that N-glycans guide partner ligands to their binding sites and prevent irregular protein aggregation by covering oligomerization sites away from the ligand-binding site [26]. Here we have shown that detailed hydropathic chain profile analysis enables higher resolution of punctuated evolution at the level of individual glycoprotein amino acids (about 20 times smaller than N-glycan spacing).

Why are proteinquakes so important at the molecular level? Formation of oncolytic core oligomers requires multiple steps. First, individual viruses must be bound to the cancer cell membrane. Next these isolated molecules must diffuse along the membrane surface to form oligomeric clusters through shear flow [27]. The surface diffusion rate over a rough surface can be accelerated by smoothing both the NA and HA glycoproteins, another reason for viral glycosidic balance [20]. Combining HA and NA into oligomeric complexes itself involves multiple conformation changes that are expected to be dominated by *π*-like sliding water package conformational dynamics, not *σ*-like backbone compression. Finally, the HA analysis leads to a block model of the HA water packaging structure that includes HA fractures (Figure 5) which could be called vaccination-driven proteinquakes. Such cumulative mechanical effects are even the cause of stiff *α* helix/soft *β* strand transitions in 56 aa proteins that share 88% sequence identity [28]. Here in 577 aa proteins the sequence identities are even higher, Rome 1949-Ft Warren1950: 94% identity (ABN59434-Q288V2).

Mechanical effects dependent on stiff/soft alternation are a common theme in structures like bone, which are strong but not brittle (high yield strength) [29]. Mechanical effects are one of the dominant factors in ligand binding [30]. The large-scale proteinquake effects described here for HA with *W* = 111 are consistent with the large-scale description of structural studies for three examples of membrane fusion that included HA and the vesicular stomatitis virus glycoprotein [14]. It is possible that quake-like unpinning effects have been observed in recent single-virus force spectroscopy (SVFS) characterizations of the interacting forces between influenza A viruses (H3N2 and H1N1) and living cells [30, 31].

Readers who wish to connect the methods used here to those of modern mathematics could consult [32], especially Sections. 7.1, 7.2 (plane and space curves), 7.3 (surfaces), 8.1 (Euclidean geometry), 8.4 (curvature), and 15.4 (expected values and variance). There are excellent Wikis on fractals and self-organized criticality. The superiority of the bioinformatic hydropathic scale [4] to the standard (>15,000 citations) water-air enthalpic scale [33] has been demonstrated for many well-studied proteins [34, 35]. The success of this historical (1945–2011) panorama depends almost entirely on the large database that has accumulated. The data have come from many sources, but the largest part of this database, especially the older parts, is due to the NIAID Influenza Genome Sequencing Project, which has proved to be invaluable.

## Figures and Tables

**Table 1:** Strain-averaged panoramic average hydropathicities 〈*ψ*〉 for the two scales in the text. The standard deviations σ for NA are taken over the panorama plateaus 1945–2011. For HA the year 1996 was used to estimate σ (see text). Also shown are lysozyme *c*, adrenergic (*β*1), and rhodopsin values for several species (lamprey, chicken, and human). In general evolution stabilizes proteins by compacting them and increasing 〈*ψ*〉. Note that rhodopsin is exceptionally stable, as it must be to receive and process optical signals. NA is noticeably hydrophilic, and HA is even more hydrophilic. The 〈*ψ*〉 trends shown here are interesting, but the opposing viral effects of migration and vaccination pressures can be recognized only in the context of the more sophisticated discussion in the text of variance (roughness).

	〈MZ〉	σ(〈MZ〉)	〈KD〉	σ(〈KD〉)
Lyso (C)	153.1			
Lyso (H)	154.7
Adren (H)	154.7
Rhodop (L)	167.1
Rhodop (C)	167.6
Rhodop (H)	167.8
NA	151.5	0.4	159.7	0.3
HA	148.9	0.6	156.5	0.7
